# Isolation of viable *Mycobacterium bovis* from faeces of naturally infected free-ranging rural domestic cattle (*Bos taurus*)

**DOI:** 10.1093/jambio/lxaf281

**Published:** 2025-11-13

**Authors:** Rachiel Gumbo, Tristen Lourens, Deborah M Cooke, Tanya J Kerr, Robin M Warren, Michele A Miller, Giovanni Ghielmetti, Wynand J Goosen

**Affiliations:** South African Medical Research Council Centre for Tuberculosis Research; Division of Molecular Biology and Human Genetics, Faculty of Medicine and Health Sciences, Stellenbosch University,Tygerberg 7505, Cape Town, South Africa; Department of Microbiology and Biochemistry, Faculty of Natural and Agricultural Sciences, University of Free State, Bloemfontein 9301, South Africa; South African Medical Research Council Centre for Tuberculosis Research; Division of Molecular Biology and Human Genetics, Faculty of Medicine and Health Sciences, Stellenbosch University,Tygerberg 7505, Cape Town, South Africa; Department of Agriculture and Rural Development, Veterinary Epidemiology, Pietermaritzburg 3202, South Africa; South African Medical Research Council Centre for Tuberculosis Research; Division of Molecular Biology and Human Genetics, Faculty of Medicine and Health Sciences, Stellenbosch University,Tygerberg 7505, Cape Town, South Africa; South African Medical Research Council Centre for Tuberculosis Research; Division of Molecular Biology and Human Genetics, Faculty of Medicine and Health Sciences, Stellenbosch University,Tygerberg 7505, Cape Town, South Africa; South African Medical Research Council Centre for Tuberculosis Research; Division of Molecular Biology and Human Genetics, Faculty of Medicine and Health Sciences, Stellenbosch University,Tygerberg 7505, Cape Town, South Africa; South African Medical Research Council Centre for Tuberculosis Research; Division of Molecular Biology and Human Genetics, Faculty of Medicine and Health Sciences, Stellenbosch University,Tygerberg 7505, Cape Town, South Africa; Section of Veterinary Bacteriology, Institute for Food Safety and Hygiene, Vetsuisse Faculty, University of Zurich, Zurich 8057, Switzerland; South African Medical Research Council Centre for Tuberculosis Research; Division of Molecular Biology and Human Genetics, Faculty of Medicine and Health Sciences, Stellenbosch University,Tygerberg 7505, Cape Town, South Africa; Department of Microbiology and Biochemistry, Faculty of Natural and Agricultural Sciences, University of Free State, Bloemfontein 9301, South Africa

**Keywords:** bovine tuberculosis, faecal shedding, GeneXpert^®^ MTB/RIF Ultra, *Mycobacterium bovis*, mycobacterial culture, nontuberculous mycobacteria

## Abstract

**Aims:**

This study investigated the presence of viable *Mycobacterium bovis* in faecal samples collected from 79 free-ranging domestic cattle in rural KwaZulu-Natal, South Africa.

**Methods and results:**

Faecal samples were processed under biosafety level 3 (BSL-3) conditions and analysed using mycobacterial culture followed by molecular speciation, as well as being screened using the GeneXpert^®^ MTB/RIF Ultra (GXU^®^) assay. Viable *M. bovis* was isolated from two animals, confirmed through region-of-difference PCR and spoligotyping. These findings provide rare field-based confirmation of natural faecal shedding of viable *M. bovis* in cattle. The GXU^®^ detected *Mycobacterium tuberculosis* complex (MTBC) DNA in eight samples (10.1%), including those that were culture positive, supporting its utility as a rapid screening tool. However, its inability to confirm bacterial viability or differentiate MTBC members remains a limitation. Additionally, nontuberculous mycobacteria (NTMs), including *M. avium* and *M. litorale*, were isolated, highlighting environmental exposure and diagnostic challenges in endemic regions.

**Conclusions:**

This study reinforces the need to consider faecal shedding and environmental reservoirs in bTB transmission dynamics, particularly in communal grazing systems. It also emphasizes the importance of integrating culture-based and molecular diagnostics for accurate detection and differentiation of mycobacterial species. These findings have important implications for One Health approaches to bTB surveillance, control, and zoonotic risk mitigation.

Impact Statement This study provides field-based evidence of viable *Mycobacterium bovis* in cattle faeces, highlighting overlooked transmission routes and reinforcing the need for integrated diagnostic approaches to improve bovine tuberculosis surveillance and control in endemic settings.

## Introduction

Bovine tuberculosis (bTB) is a chronic, infectious, and zoonotic disease caused by *Mycobacterium bovis*, a member of the *Mycobacterium tuberculosis* complex (MTBC) (Borham et al. 2022, Zhang et al. 2022). It affects a broad range of domestic and wild animals and can be transmitted to humans primarily through the inhalation of aerosols, ingestion of unpasteurized dairy products, or direct contact with infected animals (Hassan et al. 2014, Miller et al. 2015, Borham et al. 2022). The disease remains endemic in many low- and middle-income countries, particularly in sub-Saharan Africa, where the close coexistence of humans, livestock, and wildlife facilitates transmission and complicates control efforts (De Garine-Wichatitsky et al. 2013, Sichewo et al. 2019, 2020, Davey 2023). In rural settings, where veterinary infrastructure is often limited, bTB continues to have serious economic implications for livestock productivity and trade and poses significant public health risks (Mwacalimba et al. 2013, Agbalaya et al. 2020, Khairullah et al. 2024).

Traditionally, respiratory transmission has been considered the principal route for *M. bovis* spread among cattle and between animals and humans (Menzies and Neill 2000, Gannon et al. 2007, Borham et al. 2022, Khairullah et al. 2024). As such, control programmes have focused mainly on detecting pulmonary lesions and respiratory excretion of the pathogen (Palmer and Waters 2011, Pascual-Linaza et al. 2017, Jajere et al. 2018). However, emerging evidence from both experimental and observational studies suggests that *M. bovis* may also be shed through faeces, particularly in animals with advanced disease (Allen et al. 2021, Palmer et al. 2022). Faecal shedding could contribute to environmental contamination of shared water sources, soil, and grazing lands, thereby creating a potential indirect route of transmission. Despite its implications, faecal shedding of viable *M. bovis* remains underexplored and underreported under natural field conditions. Environmental reservoirs play a critical role in the persistence and transmission of *M. bovis* (Humblet et al. 2009, Dwyer et al. 2020, Matthews et al. 2025). In free-ranging livestock systems, where cattle roam and interact with wildlife, excreta such as faeces may contaminate the environment and serve as sources of infection for other animals. The potential for such transmission is particularly concerning in areas characterized by communal grazing and water use, especially where wildlife conservation and agricultural activities intersect. Investigating faecal shedding of *M. bovis* in naturally infected cattle is thus vital to fully understand the ecology of bTB, evaluate indirect transmission pathways, and refine surveillance and control strategies.

Diagnosis of *M. bovis* infection in faeces is inherently challenging due to the typically low bacillary load and the presence of complex inhibitory matrices (Allen et al. 2021, Palmer et al. 2022). Conventional methods, such as Ziehl-Neelsen (ZN) staining, suffer from low sensitivity in paucibacillary specimens and also lack specificity, as the method can only identify acid-fast bacilli without distinguishing between mycobacterial species (Coronel et al. 2019). Mycobacterial culture, although considered the gold standard, requires lengthy incubation periods and biosafety level 3 (BSL-3) facilities (Domingos et al. 2019, Thomas and Chambers 2021). Recent literature has reported that the GeneXpert^®^ MTB/RIF Ultra (GXU^®^) assay, a cartridge-based polymerase chain reaction (PCR) developed for TB diagnosis in humans, can enhance the detection of MTBC DNA in various paucibacillary samples from wildlife (Kerr et al. 2020, Goosen et al. 2020, Clarke et al. 2022). Although the GXU^®^ cannot differentiate between viable and non-viable mycobacteria, this rapid ancillary screening technique has improved the detection of MTBC infections in humans and animals (Chakravorty et al. 2017, Opota et al. 2019). However, it is important to note that in non-sterile sample types, such as faeces and environmental samples, the GXU^®^ assay may be influenced by the presence of PCR inhibitors and non-target microbial DNA, which can affect both its sensitivity and specificity. Despite these limitations, the GXU^®^ assay has demonstrated utility in detecting MTBC DNA in complex matrices (Matthews et al. 2025). Nevertheless, further validation using appropriate controls and complementary diagnostic methods is needed to ensure reliable interpretation of results. In addition, faeces may harbour nontuberculous mycobacteria (NTMs), which complicate the interpretation of immune-based diagnostics, and may be mistaken for *M. bovis* in molecular or culture assays, which could also mask the presence of MTBC (Bolaños et al. 2018, Ghielmetti et al. 2018, Clarke et al. 2022). Despite increasing attention on faecal diagnostics in wildlife and experimental cattle studies, there remains a lack of direct evidence confirming the natural shedding of viable *M. bovis* in cattle faeces under field conditions (Palmer et al. 2022). The limited data hampers accurate risk assessment and evidence-based policy formulation in high-burden, resource-limited settings.

This study aimed to investigate the feasibility of isolating viable *M. bovis* in faecal samples from free-ranging, naturally infected domestic cattle herds in rural KwaZulu-Natal, South Africa. The study area is characterized by a long-standing endemic presence of *M. bovis* infection in both domestic and wildlife populations (Hlokwe et al. 2011, Bernitz et al. 2018, Sichewo et al. 2019, 2020, Cooke et al. 2023, Mathews et al. 2025). Ongoing transmission persists in the absence of a TB surveillance programme for these populations (Davey 2023). By combining mycobacterial culture, molecular diagnostics, and species-level confirmation, we provide compelling evidence for faecal shedding of *M. bovis* under natural conditions. These findings challenge the conventional focus on respiratory transmission and underscore the importance of incorporating environmental and indirect transmission pathways into bTB control frameworks.

## Materials and methods

### Animals and faecal collection

**Figure 1.**
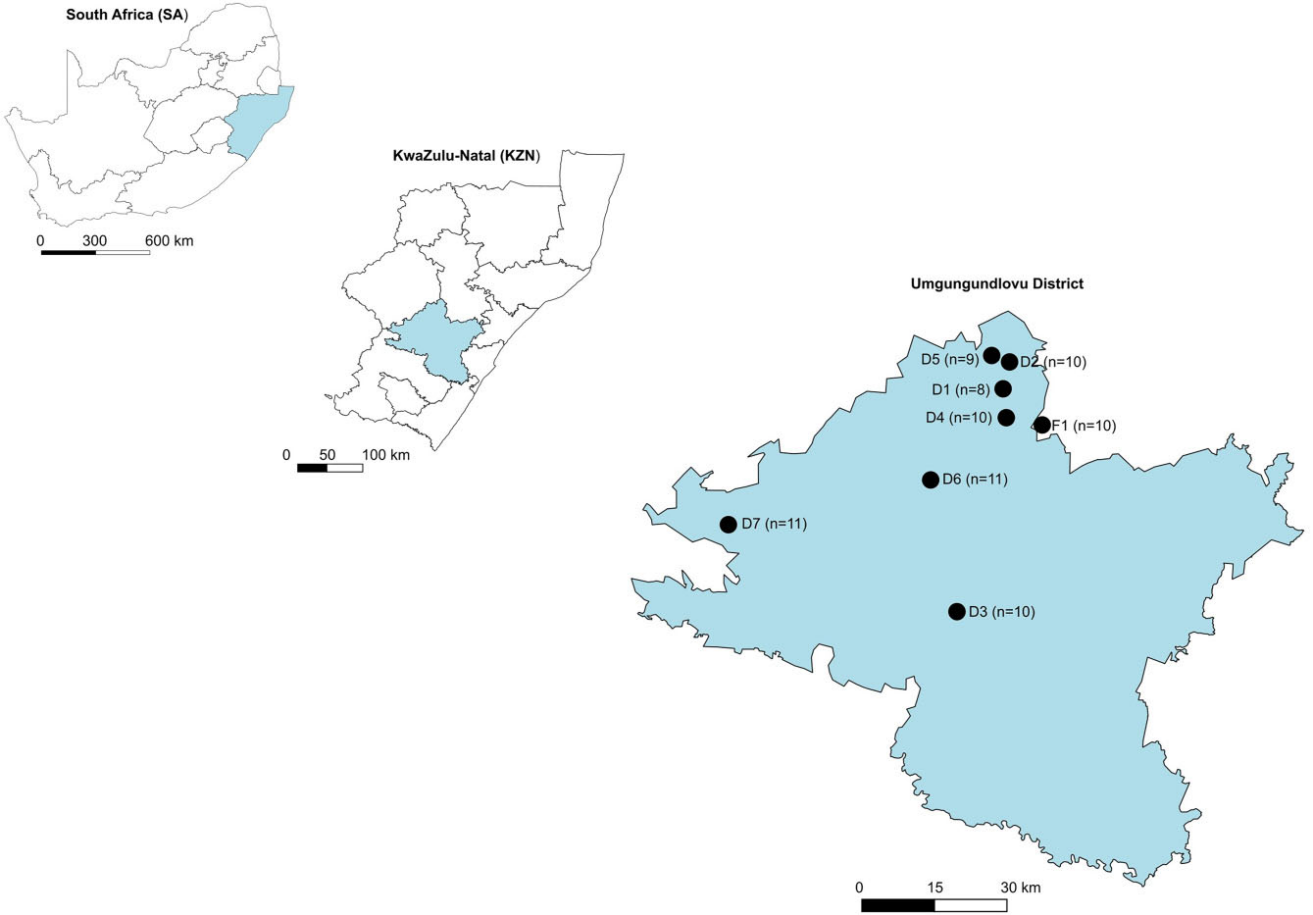
Maps showing the location of Umgungundlovu District Municipality within KwaZulu-Natal province, South Africa. Dip tanks (including one farm site) are indicated with the corresponding number of faecal samples collected at each location.

Faecal samples (50 g each, one sample per animal) were opportunistically collected from rural domestic cattle (*n* = 79) undergoing routine tick and internal parasite treatments at seven communal dip tanks and one farm in the district of Umgungundlovu situated in KwaZulu-Natal (KZN), South Africa (Fig. 1). Although consent for faecal sampling was given, owners did not provide permission for other TB testing, such as the tuberculin skin test. Faecal samples were collected over a period of 3 months between August and October 2020 and were collected directly from the rectum of animals into sealed sample containers and immediately kept cool at 4°C before being frozen at −20°C. The cattle breed, age, and sex were recorded during sample collection. Samples were later transported to an accredited laboratory, Biosafety Level 3 (BSL-3) at Stellenbosch University (SU), for further processing and analysis. The study protocol was approved by the SU Animal Care and Use Research Ethics Committee (SU-ACU-2020–15 495; SU-ACU-2021–23 032) and the Stellenbosch University Biological and Environmental Safety Research Ethics Committee (SU-BEE-2021–22 561). Section 20 approval was granted by the South African Department of Agriculture, Land Reform and Rural Development [DALRRD 12/11/1/7/2 (2700 LH); 12/11/1/7/2 (AVZ 6468)].

### Faecal processing

The pre-processing of samples was done under BSL-3 conditions. Frozen faecal samples were thawed at room temperature, and ~50 g of faecal sample from each animal was suspended in 20 ml of sterile BBL™ MycoPrep™ Phosphate Buffer (PO_4_ buffer; Becton Dickinson, Franklin Lakes, NJ, USA) in 50 ml polypropylene conical centrifuge tubes. The tubes were vortexed at maximum speed and centrifuged at 800 × *g* for 10 minutes using Eppendorf Ultra Centrifuge 5810 R (Eppendorf SE, Barkhausenweg 1, Hamburg, Germany). While avoiding debris, the supernatant was harvested into new polypropylene 50 ml conical centrifuge tubes. The new tubes were centrifuged at 3000 × *g* for 20 minutes. The supernatant was discarded, and the pellet was resuspended in 4 ml of PO_4_ buffer. Approximately 2 ml of the suspension was aliquoted into 2 ml microcentrifuge tubes and heat-inactivated at 100°C for 30 minutes for the GeneXpert^®^ MTB/RIF Ultra (GXU) qPCR assay (Cepheid Inc., Sunnyvale, CA, USA), performed under biosafety level 2 conditions.

### Mycobacterial culture

The remainder of the suspension (approximately 2 ml) in the 50 ml conical centrifuge tubes was decontaminated using 2.5 ml of BD BBL™ MycoPrep™ N-acetyl-L-cysteine and sodium hydroxide (NALC-NaOH; Becton Dickinson). After the addition of NALC-NaOH solution, samples were vortexed briefly, incubated for 15 minutes at room temperature with continued mixing by vortexing at regular intervals during the incubation period to allow adequate decontamination. Thereafter, samples were neutralized with sterile 15 ml PO_4_ buffer and vortexed thoroughly. Samples were centrifuged for 20 minutes at 3000 × *g* before discarding the supernatant. Each pellet was resuspended in 500 µl sterile PO_4_ buffer before transferring all the contents into Mycobacteria Growth Indicator Tubes (MGIT™; Becton Dickinson). Each MGIT™ tube was supplemented with 800 μl of BD BACTEC™ MGIT™ 960 Supplement kit (BBL MGIT™ PANTA antimicrobial agent mixture reconstituted with 15 ml of Oleic Acid-Albumin-Dextrose-Catalase (OADC)) and 100 µl of 4.2 g/l sodium pyruvate (Sigma–Aldrich, St. Louis, MO, USA). The MGIT™ tubes were incubated for 56 days in a BACTEC™ MGIT™ 960 Mycobacterial Detection System (Becton Dickinson) at 37°C.

**Figure 2.**
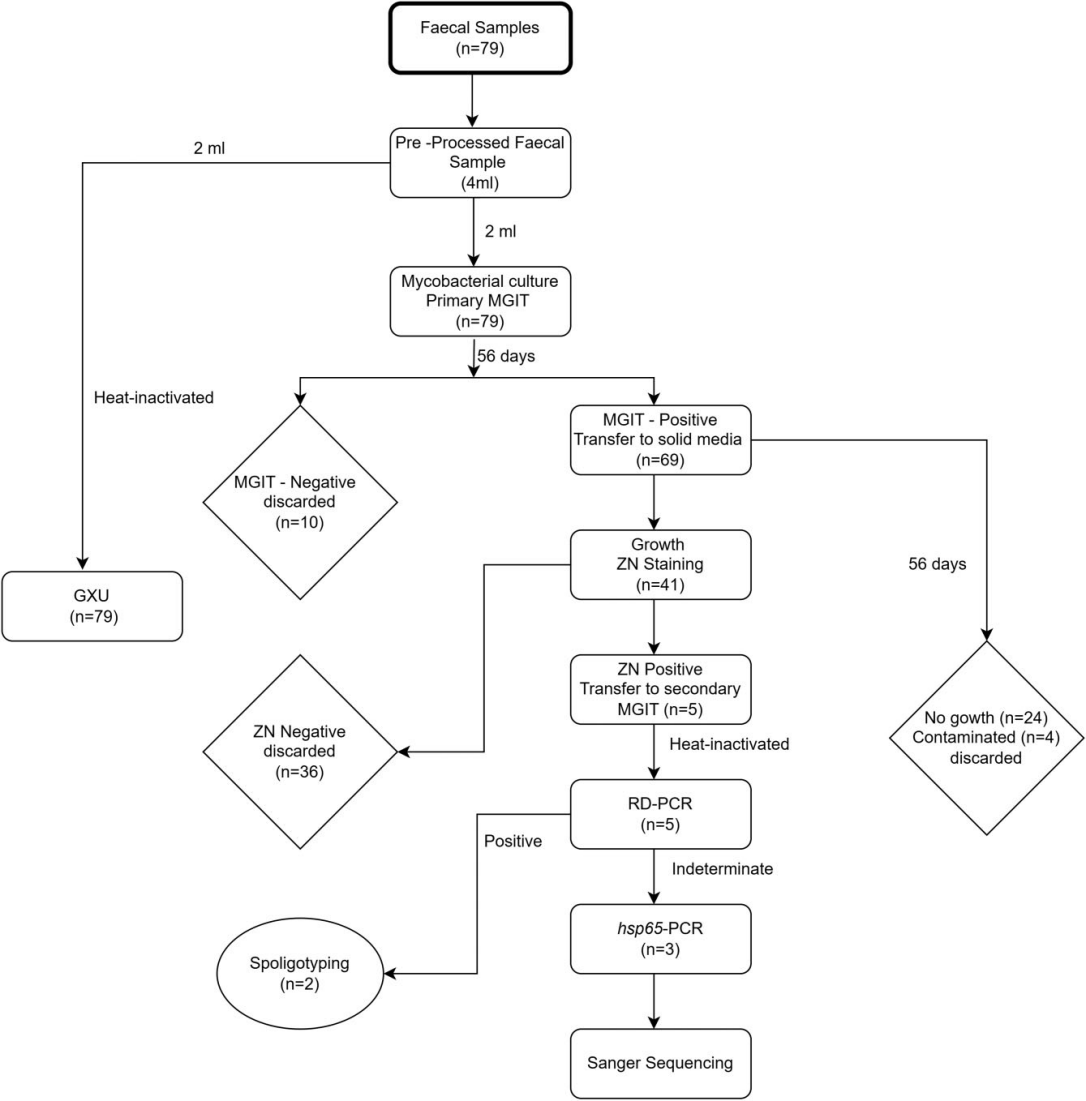
Study overview illustrating the steps followed during the process of cattle faecal samples. The flowchart includes sample processing, mycobacterial culture (liquid and solid), Ziehl-Neelsen staining, polymerase chain reaction, Sanger sequencing, and GeneXpert^®^ MTB/RIF Ultra qPCR (GXU^®^).

After 56 days, the MGIT™ tubes that were negative according to the BACTEC™ MGIT™ 960 Mycobacterial Detection System were discarded. For tubes that flagged positive, 35 µl from the bottom of the MGIT™ culture tubes were inoculated onto solid media using Pasteur pipettes (Fig. 2). Solid media preparation was performed as follows. Briefly, 9.5 g of Difco™ Middlebrook 7H10 agar base (Becton Dickinson) was mixed with 2.1 g sodium pyruvate (Sigma–Aldrich), 0.5 g tryptone (Sigma–Aldrich), and 2.5 ml of glycerol (Sigma–Aldrich) in 450 ml of distilled water. All components were thoroughly mixed both before and after sterilization at 121°C for 20 minutes. The mixture was subsequently cooled to 55°C, followed by the addition of 60 ml (4 bottles) of reconstituted PANTA-OADC enrichment (Becton Dickinson) before immediately pouring approximately 20 ml into each Petri dish. Following inoculation, plates were incubated at 37°C and the growth of colonies was checked every 2 weeks for a period of 8 weeks. Upon detection of bacterial growth, Ziehl-Neelsen (ZN) staining was performed on all single colonies from all plates exhibiting growth. Plates with all colonies that were ZN negative were discarded after 56 days of incubation. ZN-positive colonies were subcultured into secondary MGIT™ tubes and incubated for 2 weeks in a BACTEC™ MGIT™ 960 machine (Fig. 2). After 2 weeks, if the secondary MGIT™ tubes flagged positive, an aliquot from each tube was heat-inactivated, and bacterial isolates were genetically speciated by genomic regions of difference (RD) PCR (Warren et al. 2006) and spoligotyping (Kamerbeek et al. 1997). The spoligotype of the isolates from this study was authenticated by comparing the observed spoligotype pattern to known spoligotypes in the *Mycobacterium bovis* Spoligotype Database (https://www.mbovis.org/database.php) (Smith and Upton 2012).

Samples that had indeterminate results with RD-PCR were screened using primers targeting the heat shock protein of the 65-kDa (*hsp65*) gene (Telenti et al. 1993). The PCR was performed by combining 2 µl of the secondary MGIT™ contents with 12.5 µl OneTaq^®^ Hot Start 2X Master Mix with Standard Buffer (New England Biolabs, Ipswich, MA, USA), 9.5 µl nuclease-free water (Ambion, Austin, TX, USA), and 0.5 µl (10 µM) of each primer (Integrated DNA Technologies, Coralville, IA, USA) in a final volume of 25 µl. Reactions were initiated at 94°C for 10 minutes, followed by 40 cycles of denaturation at 94°C for 30 seconds, annealing at 62.5°C for 30 seconds, and extension at 68°C for 30 seconds, concluding with a final extension at 72°C for 5 minutes, using an Applied Biosystems Veriti™ Thermal Cycler (ThermoFisher Scientific, Wilmington, DE, USA). To confirm amplification and band sizes, PCR products were visualized using a 1% agarose gel in sodium borate (SB) buffer (Sigma–Aldrich), after which samples were sent to the Stellenbosch University Central Analytical Facility (CAF, Stellenbosch, South Africa) for post-PCR clean-up and Sanger sequence confirmation. Sequences were aligned and edited using ApE (version 3.1.7, University of Utah, Salt Lake City, UT, USA) before employing the National Centre for Biotechnology Information (NCBI) Basic Local Alignment Search Tool (BLAST) (https://blast.ncbi.nlm.nih.gov) software to compare these nucleotide sequences with available sequence databases. Sequences were identified based on a minimum of 80% coverage and sequence identity thresholds of ≥97% for genus and ≥99% for species-level classification. Isolates identified as members of the MAC based on *hsp65* Sanger sequencing results using the above-mentioned primers underwent amplification and sequencing of the entire *hsp65* gene, following the methodology described by Turenne et al. (2006).

### GeneXpert^®^ MTB/RIF Ultra qPCR assay (GXU^®^)

The GXU^®^ is a rapid ancillary method to mycobacterial culture that was used in this study to confirm the presence of MTBC DNA directly in cattle faecal suspensions that were initially heat-inactivated in BSL3. Briefly, 2.0 ml of the GXU^®^ Sample Lysis Reagent (Cepheid) was added to each 1.0 ml of faecal suspension. Samples were vortexed thoroughly and incubated for 15 minutes according to Chakravorty et al. (2017). After incubation, 2 ml was transferred to a GXU^®^ cartridge (Cepheid) and tested using the Cepheid GeneXpert^®^ Instrument system. Results for GXU^®^ were categorized as follows: (a) MTBC not detected; (b) MTBC trace detected; (c) MTBC detected high/medium/low/very low, based on the presence of the IS*6110* and/or IS*1081* molecular signals (Chakravorty et al. 2017). A result was considered positive for MTBC if any category other than “MTBC not detected” was reported.

### Statistical analysis

Proportions of GXU^®^ test-positive individuals were compared across sex and age categories using Fisher’s exact test. A *P*-value < 0.05 was considered statistically significant. All statistical analyses were performed using GraphPad Prism 10 for Windows (version 10.2, GraphPad Software, Inc., San Diego, CA, USA; www.graphpad.com).

## Results

All rural free-ranging communal cattle included in this study, except one with unknown breed information, were Nguni crossbreeds (Supplementary Table S1). The study comprised 64 females and 14 males, of which 70 were adults, 8 sub-adults, and 1 individual for which sex and age not recorded (Table 1). All samples were opportunistically collected for purposes unrelated to this study; therefore, clinical data for these animals were unavailable.

**Table 1.** Summary of demographic information, GXU^®^ and mycobacterial culture results for faeces collected from free-ranging rural domestic cattle (*n* = 79) at multiple dip tanks and a farm in KwaZulu-Natal, South Africa.

	Sex		Age		GXU^®^ results		Mycobacterial culture results			
	Males	Females	Adults	Sub-adults	MTBC detected	MTBC not detected	MGIT^™^ positive	ZN Positive	NTMs isolated	*M. bovis* isolated
F1	1	9	7	3	3	7	6	3	1	2
D1	4	4	8	0	4	4	3	1	1	0
D2	1	9	10	0	0	10	10	0	0	0
D3	3	7	8	2	1	9	10	0	0	0
D4	1	9	10	0	0	10	10	1	1	0
D5	0	9	9	0	0	9	8	0	0	0
D6	0	11	10	1	0	11	11	0	0	0
D7	4	6	8	2	0	11	11	0	0	0
**Total**	**14**	**64**	**70**	**8**	**8**	**71**	**69**	**5**	**3**	**2**

GXU^®^: GeneXpert^®^ MTB/RIF Ultra; MTBC: *Mycobacterium tuberculosis* Complex; MGIT™: Mycobacteria Growth Indicator Tubes; ZN: Ziehl-Neelsen; NTMs: Nontuberculous mycobacteria; *M. bovis: Mycobacteria bovis*. Speciation of ZN-positive cultures was achieved by region of difference (RD) and heat shock protein of the 65-kDa (*hsp65*) PCR analysis.

From the 79 faecal samples that were inoculated into MGIT™ tubes, 10/79 (12.7%) samples were MGIT™ negative and were discarded, while 69/79 (87.3%) flagged positive on the MGIT™ 960 Mycobacterial Detection System (Fig. 2; Table 1). Of the 69 MGIT™ tubes that were subcultured on solid media, 41 (59.4%) showed growth of bacterial colonies and were stained using ZN, 24 (34.8%) had no growth after 8 weeks of incubation, while 4 (5.8%) were discarded due to high contamination rates with non-acid-fast bacteria or mould (Fig. 2). Among the 41 plates with growth, 36/41 (87.8%) were ZN-negative and were discarded, while RD-PCR was performed on 5/41 (12.2%) ZN-positive isolates (Table 1).

*Mycobacterium bovis* was confirmed by RD-PCR in 2/5 (40%) of the ZN-positive isolates (WG31 and WG34; Supplementary Table S1). The strain isolated from both individuals was spoligotype SB0130 (Supplementary Table S1). *Mycobacterium* species and *Mycobacterium litorale* (99.1% identity match (IM)) were isolated from WG20 and WG64, respectively (Supplementary Table S1). In addition, *Mycobacterium avium* complex (99.3% IM), was isolated from WG25 (Supplementary Table S1). *Mycobacterium avium* subspecies *paratuberculosis* (MAP) was excluded based on sequencing of the complete *hsp65* gene, followed by comparative analysis using NCBI BLAST to confirm the absence of MAP-specific sequence identity.

**Figure 3.**
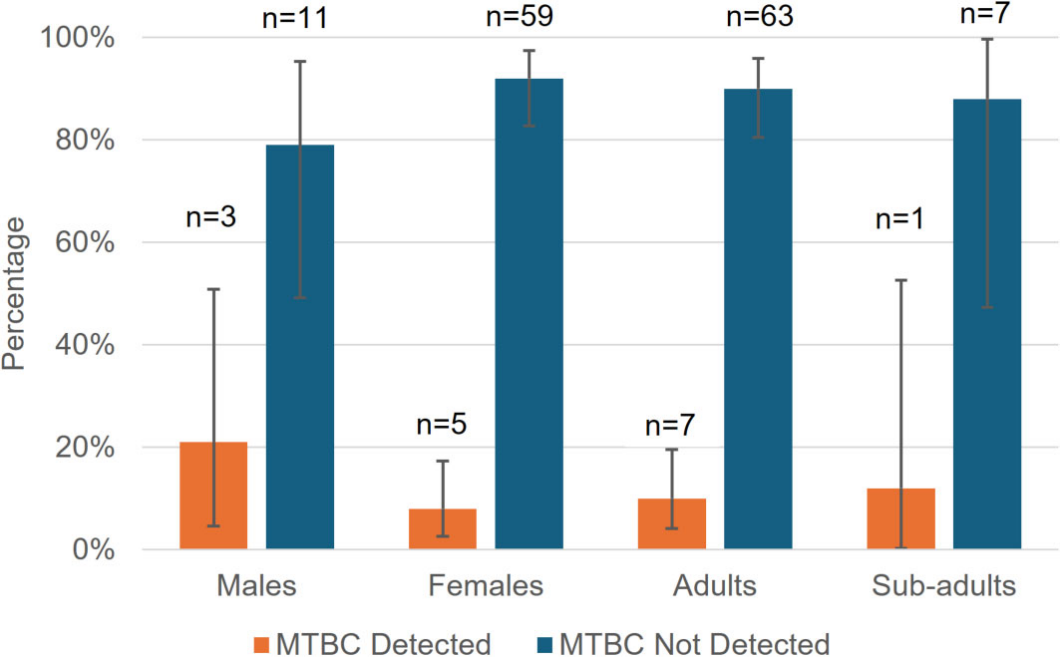
Clustered bar graph showing the proportion of cattle faeces testing positive for *Mycobacterium tuberculosis* complex (MTBC) DNA by sex and age class as determined using GeneXpert^®^ MTB/RIF Ultra qPCR assay. Error bars indicate the 95% confidence intervals for the proportion estimates.

Using the GXU^®^ assay, MTBC DNA was detected in 8/79 faecal samples (10%) and was categorized as trace (*n* = 4), low (*n* = 1), and high (*n* = 3), including the two samples from which viable *M. bovis* was isolated. In addition, the proportion of individuals with detectable MTBC DNA was highest among males (3/14; 21%) compared to females (5/64; 8%) (Fig. 3), although this difference was not statistically significant (*P* = 0.15). Out of 8 sub-adults, only one animal (12%) had MTBC DNA detected, while adults recorded 7/70 (10%) (Fig. 3), with no significant difference observed between the groups (*P* > 0.99).

Dip tank D1 recorded the highest number of MTBC DNA-positive animals (*n* = 4) using the GXU^®^ (Table 1). Notably, three of these individuals were males (WG15, WG16, and WG18; Supplementary Table S1). However, no growth of mycobacteria was observed from any of the three males using both liquid and solid media. At the same time, the female animal (WG20) was GXU^®^ positive (trace) for MTBC DNA and *Mycobacterium* species was isolated (Supplementary Table S1). The *M. bovis* isolates were cultured from one adult and one sub-adult female cow, both from farm F1. These individuals were also GXU^®^ positive for MTBC DNA, in their faeces (low and high). In addition, *M. avium* was isolated from WG25, a cow that was negative for MTBC DNA (GXU^®)^ (Supplementary Table S1).

## Discussion

This study reports the successful isolation of viable *M. bovis* from faecal samples of two naturally infected free-ranging communal domestic cattle, providing direct evidence of faecal shedding as a potential pathway for environmental contamination and disease transmission. The results also demonstrated the potential diagnostic use of the GXU^®^ assay for detecting MTBC DNA in cattle faeces, offering rapid and reliable results in a significantly shorter time than mycobacterial culture. Additionally, the isolation of nontuberculous mycobacteria such as *M. avium* and *M. litorale* from cattle faeces has important diagnostic, epidemiological, and ecological implications.

Of the 79 faecal samples inoculated into primary MGIT™ tubes, 87.3% flagged positive, detecting bacterial and fungal growth. However, only 41 of the 69 primary MGIT™ tubes subcultured onto solid media produced visible bacterial colonies. This could be due to the presence of aerobic bacteria (non-mycobacterial) or moulds that had survived the decontamination process, overgrown the targeted mycobacteria in the liquid medium, therefore, consuming oxygen and causing the MGIT™ tubes to flag positive, but still their growth was not supported by the solid media (Mahomed et al. 2017). Lack of colony formation on solid media may also be due to the presence of non-viable mycobacteria or low bacterial load. This is insufficient for colony formation, even though fluorescence was triggered in liquid culture. Additionally, certain NTMs, especially slow-growing or fastidious strains, possess unique nutritional or environmental requirements that are not supported by the solid media, potentially leading to a lack of visible colony formation (Chew et al. 1998, Hines et al. 2006, Bittner and Preheim 2016). The detection of MGIT™ positive cultures without corresponding acid-fast bacilli on microscopy is consistent with the presence of non-mycobacterial contaminants, which may have triggered false-positive growth signals.

*Mycobacterium bovis* was isolated from two female cows located on the same farm and these isolates shared the same spoligotype (SB0130). This suggests either a common source of infection, such as contaminated feed or water or localized transmission dynamics (Sichewo et al. 2020, Szacawa et al. 2025). Both cows also tested positive for faecal MTBC DNA, supporting the utility of GXU^®^ as a rapid screening tool for complex specimens. However, the inability of GXU^®^ to distinguish between viable and non-viable mycobacteria, and to differentiate between members of MTBC remains a major limitation. A possible limitation of the GXU^®^ is the detection of environmental NTM DNA, which could potentially contribute to false-positive MTBC results. For instance, in sample WG20, MTBC DNA was detected, but culture identified a *Mycobacterium* species but not MTBC, raising the possibility that environmental NTM may have influenced the assay outcome. In addition, the detection of MTBC DNA from five other individuals that did not produce viable cultures indicated shedding of non-viable mycobacteria or low bacillary load, which is below the limit of detection of our mycobacterial culture protocol. However, the vigorous decontamination process before mycobacterial culture may have also contributed to no growth of MTBC from faeces of WG15, WG16, WG18, WG28, and WG52 (Goosen et al. 2022). The application of GXU^®^ for the detection of MTBC DNA in both domestic and wild animals has been previously documented, emphasizing its high sensitivity, particularly in paucibacillary samples (Goosen et al. 2020, Ntivuguruzwa et al. 2022, Boko et al. 2022, Cooke et al. 2023, Gumbo et al. 2023).

In addition to *M. bovis*, the isolation of NTMs such as *M. avium* and *M. litorale* in this study carries important epidemiological, diagnostic, and public health implications. *Mycobacterium avium* complex (MAC) comprises a group of closely related, established environmental opportunistic pathogens that are commonly present in various environmental conditions such as soil and water sources (Pavlik et al. 2005, Rindi and Garzelli 2014). They are known to cause a spectrum of diseases in both animals and humans, including tuberculosis-like diseases in immunocompromised individuals (Thorel et al. 2001, Field et al. 2004, Griffith et al. 2007). The isolation of MAC in this study reflects exposure to a contaminated environment. These infections present significant diagnostic challenges in bTB surveillance programmes (Barry et al. 2011). Cattle exposed to *M. avium* subspecies *avium* and *paratuberculosis* can elicit cross-reactive immune responses in intradermal tuberculin skin tests (TST) and interferon-gamma release assays (IGRAs) (Barry et al. 2011, Gormley et al. 2013, Jones et al. 2017). However, in this study, based on *hsp65*-PCR, the presence of MAP was excluded.

Although the pathogenic potential of M. *litorale* in cattle remains unclear, the possible isolation of NTMs from cattle faeces in this study should be interpreted with caution, since these may represent GI passage from environmental sources (Zulu et al. 2021). Notably, the presence of diverse mycobacterial species isolated from cattle populations emphasizes the necessity for robust differential diagnostic approaches, which are essential for distinguishing *M. bovis* from non-pathogenic or opportunistic NTMs, especially in cultures from faecal samples. Ideally, sequencing additional targets such as the DNA-directed RNA polymerase subunit beta (*rpoB*) and 16S ribosomal RNA (16S rRNA) in this study, would have provided greater taxonomic resolution. The coexistence of zoonotic and environmental mycobacteria highlights the complex interconnectedness between animal, environmental, and human health. This complexity reinforces the importance of adopting a comprehensive One Health strategy for the effective surveillance and management of mycobacterial diseases.

Although this study confirmed only two *M. bovis* cases despite high MGIT™ positivity, the sex and age distributions of GXU^®^-positive cases provide additional epidemiological insights. The higher total number of MTBC DNA-positive results in females and adults, despite the absence of viable cultures, could reflect differences in immunological responses, environmental exposure, or behavioural factors (Brooks-Pollock et al. 2013, Katale et al. 2013, Broughan et al. 2016). Though the small sample size limits definitive conclusions, our findings align with previous observations indicating that older individuals are more likely to be infected, due to cumulative exposure over time and age-related changes in immune function (Holder et al. 2024). Additionally, cattle owners’ preferences may also contribute to this pattern, as females are typically retained in herds longer than males due to their roles in reproduction and milk production (Agbalaya et al. 2020).

The successful isolation of viable *M. bovis* from samples of infected free-ranging domestic or wild animals represents a significant advance in understanding the potential role of environmental contamination in disease transmission in both rural settings and wildlife parks. Based on our knowledge, this is the first study in which viable *M. bovis* shed in the faeces of naturally infected cattle has been cultured. It is essential to recognize that the detection of culturable bacilli indicates that infected animals have developed sufficient disease to permit bacterial shedding, although passive passage from a contaminated environment cannot be ruled out. Our findings reinforce concerns about the role of faecal-oral and environmental transmission pathways in the epidemiology of bTB (Barbier et al. 2017, Pereira et al. 2024). In addition, sequencing of cultured isolates facilitates the identification of epidemiologic links (Pereira et al. 2024). Isolation of *M. bovis* from faeces challenges the traditional view that respiratory routes are the primary mode of transmission. It suggests that gastrointestinal involvement and shedding may be more common in natural infections than previously recognized (Sweeney et al. 2007). It is also important to consider the geographical context of this study, as the dip tanks where these cattle were managed are located in KwaZulu-Natal province, a region with documented cases of *M. bovis* infections in both wildlife and domestic animals (Hlokwe et al. 2011, Sichewo et al. 2020, Matthews et al. 2025).

While faecal sampling offers a non-invasive and practical approach for evaluating transmission risks in shared environments, its diagnostic utility may be constrained by reduced sensitivity, mainly due to the intermittent shedding of mycobacteria in faeces. However, recent advances in molecular diagnostics, such as DNA extraction protocols incorporating bead beating and magnetic-particle isolation, have significantly enhanced the recovery of mycobacterial DNA from faecal samples (Plain et al. 2015). Additionally, highly sensitive techniques like digital droplet PCR (ddPCR) and optimized quantitative PCR (qPCR) protocols have demonstrated improved detection limits and reproducibility, offering promising solutions to overcome the limitations of conventional methods, while RNA-based molecular diagnostics may further enhance sensitivity by enabling the detection of viable bacteria (Li et al. 2024).

Future studies should aim to characterize the specific *M. avium* subspecies present and assess their virulence, environmental persistence, and potential zoonotic risk in cattle-rearing systems. Further investigation is required to assess whether the sample processing workflow, including aggressive pre-treatment and decontamination, as well as the sample handling protocol, specifically the lack of controlled, gradual cooling prior to freezing at –20°C contributes to the lack of mycobacterial growth on solid media.

## Conclusion

This study provides novel evidence of faecal shedding of viable *M. bovis* in naturally infected, free-ranging domestic cattle, highlighting the potential role of the gastrointestinal route in the transmission and environmental dissemination of bTB. The detection of *M. bovis* in faeces, supported by spoligotyping and GXU^®^ assay results, highlights the value of non-invasive sampling and rapid molecular diagnostics for surveillance. However, limitations in culture sensitivity, likely due to intermittent shedding, low bacillary load, or sample contamination effects, remain important constraints. The isolation of diverse NTMs highlights the need for sensitive molecular diagnostics and a One Health approach to improve mycobacterial disease surveillance and control across livestock, wildlife, and environmental interfaces.

## Supplementary Material

lxaf281_Supplemental_File

## Data Availability

Data available on request.
