# Supplementary material for: Isolation of viable Mycobacterium bovis from faeces of naturally infected free-ranging rural domestic cattle (Bos taurus)
Source: J Appl Microbiol. 2025 Nov 13;136(12):lxaf281. doi: 10.1093/jambio/lxaf281 (PMC12661519; doi:10.1093/jambio/lxaf281)
Supplement: lxaf281_Supplemental_File [file lxaf281_supplemental_file.docx]

**Supplementary Table 1:** Demographic information for free-ranging rural domestic cattle (n = 79) sampled from KwaZulu-Natal, South Africa, showing faecal GeneXpert^®^ MTB/RIF Ultra, mycobacterial culture, and speciation (using region of difference and heat shock protein of the 65-kDa PCR) results.

| **ID** | **Cattle Breed** | **Age** | **Sex** | **Dip tank/Farm** | **GXU^®^** | **Mycobacterial Culture and Speciation** |
| --- | --- | --- | --- | --- | --- | --- |
| WG13 | Nguni mix | Adult | Male | D1 | MTBC Not Detected | MGIT™ negative |
| WG15 | Nguni mix | Adult | Male | D1 | MTBC Detected - High | No growth |
| WG16 | Nguni mix | Adult | Male | D1 | MTBC Detected - Trace | MGIT™ negative |
| WG17 | Nguni mix | Adult | Female | D1 | MTBC Not Detected | MGIT™ negative |
| WG18 | Nguni mix | Adult | Male | D1 | MTBC Detected - Trace | MGIT™ negative |
| WG19 | Nguni mix | Adult | Female | D1 | MTBC Not Detected | No growth |
| WG20 | Nguni mix | Adult | Female | D1 | MTBC Detected - Trace | *Mycobacterium* species |
| WG24 | Nguni mix | Adult | Female | D1 | MTBC Not Detected | MGIT™ negative |
| WG25 | Nguni mix | Adult | Female | F1 | MTBC Not Detected | *Mycobacterium avium* |
| WG26 | Nguni mix | Adult | Female | F1 | MTBC Not Detected | MGIT™ negative |
| WG27 | Nguni mix | Adult | Male - Ox | F1 | MTBC Not Detected | MGIT™ negative |
| WG28 | Nguni mix | Adult | Female | F1 | MTBC Detected - High | MGIT™ negative |
| WG29 | Nguni mix | Sub-Adult | Female | F1 | MTBC Not Detected | MGIT™ negative |
| WG30 | Nguni mix | Adult | Female | F1 | MTBC Not Detected | No growth |
| WG31 | Nguni mix | Sub-Adult | Female | F1 | MTBC Detected - Low | *Mycobacterium bovis* – SB0130 |
| WG32 | Nguni mix | Heifer | Female | F1 | MTBC Not Detected | No growth |
| WG33 | Nguni mix | Adult | Female | F1 | MTBC Not Detected | Contaminated |
| WG34 | Nguni mix | Adult | Female | F1 | MTBC Detected - High | *Mycobacterium bovis* – SB0130 |
| WG35 | Nguni mix | Adult | Female | D2 | MTBC Not Detected | ZN negative |
| WG36 | Nguni mix | Adult | Male | D2 | MTBC Not Detected | ZN negative |
| WG37 | Nguni mix | Adult | Female | D2 | MTBC Not Detected | ZN negative |
| WG38 | Nguni mix | Adult | Female | D2 | MTBC Not Detected | ZN negative |
| WG39 | Nguni mix | Adult | Female | D2 | MTBC Not Detected | ZN negative |
| WG40 | Nguni mix Brahman | Adult | Female | D2 | MTBC Not Detected | ZN negative |
| WG42 | Nguni mix | Adult | Female | D2 | MTBC Not Detected | ZN negative |
| WG43 | Nguni mix | Adult | Female | D2 | MTBC Not Detected | ZN negative |
| WG44 | Nguni mix | Adult | Female | D2 | MTBC Not Detected | ZN negative |
| WG45 | Nguni mix | Adult | Female | D3 | MTBC Not Detected | ZN negative |
| WG46 | Nguni mix | Sub adult | Male | D3 | MTBC Not Detected | No growth |
| WG47 | Nguni mix Brahman | Sub adult | Male | D3 | MTBC Not Detected | No growth |
| WG49 | Nguni mix | Adult | Female | D3 | MTBC Not Detected | No growth |
| WG50 | Nguni mix | Adult | Female | D3 | MTBC Not Detected | No growth |
| WG51 | Nguni mix | Adult | Female | D3 | MTBC Not Detected | ZN negative |
| WG52 | Nguni mix | Adult | Female | D3 | MTBC Detected - Trace | No growth |
| WG54 | Nguni mix | Adult | Female | D3 | MTBC Not Detected | No growth |
| WG55 | Nguni mix | Adult | Female | D4 | MTBC Not Detected | ZN negative |
| WG56 | Nguni mix | Adult | Female | D4 | MTBC Not Detected | ZN negative |
| WG57 | Nguni mix | Adult | Male | D4 | MTBC Not Detected | No growth |
| WG58 | Nguni mix | Adult | Female | D4 | MTBC Not Detected | No growth |
| WG60 | Nguni mix | Adult | Female | D4 | MTBC Not Detected | ZN negative |
| WG61 | Nguni mix | Adult | Female | D4 | MTBC Not Detected | ZN negative |
| WG62 | Nguni mix | Adult | Female | D4 | MTBC Not Detected | ZN negative |
| WG63 | Nguni mix | Adult | Female | D4 | MTBC Not Detected | Contaminated |
| WG64 | Nguni mix | Adult | Female | D4 | MTBC Not Detected | *Mycobacterium litorale* |
| WG65 | Nguni mix Brahman | Adult | Female | D5 | MTBC Not Detected | No growth |
| WG67 | Nguni mix Brahman | Adult | Female | D5 | MTBC Not Detected | ZN negative |
| WG68 | Nguni mix Brahman | Adult | Female | D5 | MTBC Not Detected | ZN negative |
| WG69 | Nguni mix Brahman | Adult | Female | D5 | MTBC Not Detected | No growth |
| WG70 | Nguni mix Brahman | Adult | Female | D5 | MTBC Not Detected | MGIT™ negative |
| WG71 | Nguni mix Brahman | Adult | Female | D5 | MTBC Not Detected | ZN negative |
| WG73 | Nguni mix Brahman | Adult | Female | D5 | MTBC Not Detected | ZN negative |
| WG75 | Nguni mix | Adult | Female | D6 | MTBC Not Detected | ZN negative |
| WG76 | Nguni mix | Adult | Female | D6 | MTBC Not Detected | ZN negative |
| WG77 | Nguni mix | Adult | Female | D6 | MTBC Not Detected | ZN negative |
| WG78 | Nguni mix | Sub Adult | Female | D6 | MTBC Not Detected | ZN negative |
| WG79 | Nguni mix | Adult | Female | D6 | MTBC Not Detected | ZN negative |
| WG80 | Nguni mix | Adult | Female | D6 | MTBC Not Detected | Contaminated |
| WG81 | Nguni mix | Adult | Female | D6 | MTBC Not Detected | No growth |
| WG82 | Nguni mix | Adult | Female | D6 | MTBC Not Detected | ZN negative |
| WG83 | Nguni mix | Adult | Female | D6 | MTBC Not Detected | Contaminated |
| WG84 | Nguni mix | Adult | Female | D6 | MTBC Not Detected | ZN negative |
| WG85 | Nguni mix | Adult | Female | D7 | MTBC Not Detected | No growth |
| WG87 | Nguni mix | Adult | Female | D7 | MTBC Not Detected | ZN negative |
| WG88 | Nguni mix | Adult | Female | D7 | MTBC Not Detected | ZN negative |
| WG89 | Nguni mix | Sub Adult | Male - Ox | D7 | MTBC Not Detected | No growth |
| WG90 | Nguni mix | Adult | Male - Ox | D7 | MTBC Not Detected | ZN negative |
| WG91 | Nguni mix | Adult | Female | D7 | MTBC Not Detected | ZN negative |
| WG93 | Nguni mix | Sub Adult | Male - Ox | D7 | MTBC Not Detected | ZN negative |
| WG95 | Nguni mix | Adult | Male - Ox | D7 | MTBC Not Detected | No growth |
| WG96 | Nguni mix | Adult | Female | D2 | MTBC Not Detected | ZN negative |
| WG97 | Nguni mix | Adult | Female | D3 | MTBC Not Detected | No growth |
| WG98 | Nguni mix | Adult | Male | D3 | MTBC Not Detected | No growth |
| WG99 | Nguni mix | Adult | Female | D4 | MTBC Not Detected | ZN negative |
| WG100 | Nguni mix Brahman | Adult | Female | D5 | MTBC Not Detected | No growth |
| WG101 | Nguni mix Brahman | Adult | Female | D5 | MTBC Not Detected | ZN negative |
| WG102 | Nguni mix | Adult | Female | D6 | MTBC Not Detected | No growth |
| WG103 | Nguni mix | Adult | Female | D7 | MTBC Not Detected | ZN negative |
| WG104 | Unknown | Unknown | Unknown | D7 | MTBC Not Detected | No growth |
| WG105 | Nguni mix | Adult | Female | D7 | MTBC Not Detected | No growth |
| ID: Cattle Identification Number; GXU^®^: GeneXpert^®^ MTB/RIF Ultra; MTBC: *Mycobacterium tuberculosis* complex; MGIT™: Mycobacteria Growth Indicator Tubes; MGIT™ Negative was interpreted as no growth in liquid culture; No growth was interpreted as no growth on solid media; ZN: Ziehl-Neelsen; Contaminated was interpreted as growth of non-acid-fast bacteria or moulds. | | | | | | |
